# Prognostic value of circulating markers of neutrophil activation, neutrophil extracellular traps, coagulation and fibrinolysis in patients with terminal cancer

**DOI:** 10.1038/s41598-021-84476-3

**Published:** 2021-03-03

**Authors:** Axel Rosell, Katherina Aguilera, Yohei Hisada, Clare Schmedes, Nigel Mackman, Håkan Wallén, Staffan Lundström, Charlotte Thålin

**Affiliations:** 1 Department of Clinical Sciences, Danderyd Hospital, Division of Internal Medicine, Karolinska Institutet, Stockholm, 182 88 Sweden; 2 UNC Blood Research Center, Division of Hematology, Department of Medicine, University of North Carolina at Chapel Hill, Chapel Hill, NC USA; 3 Department of Clinical Sciences, Danderyd Hospital, Division of Cardiovascular Medicine, Karolinska Institutet, Stockholm, Sweden; 4 Palliative Care Services and R&D-Unit, Stockholms Sjukhem Foundation, Stockholm, Sweden; 5 Department of Oncology-Pathology, Karolinska Institutet, Stockholm, Sweden

**Keywords:** Cancer, Oncology, Immunology, Coagulation system

## Abstract

Predicting survival accurately in patients with advanced cancer is important in guiding interventions and planning future care. Objective tools are therefore needed. Blood biomarkers are appealing due to their rapid measurement and objective nature. Thrombosis is a common complication in cancer. Recent data indicate that tumor-induced neutrophil extracellular traps (NETs) are pro-thrombotic. We therefore performed a comprehensive investigation of circulating markers of neutrophil activation, NET formation, coagulation and fibrinolysis in 106 patients with terminal cancer. We found that neutrophil activation and NET markers were prognostic in terminal cancer patients. Interestingly, markers of coagulation and fibrinolysis did not have a prognostic value in this patient group, and there were weak or no correlations between these markers and markers of neutrophil activation and NETs. This suggest that NETs are linked to a poor prognosis through pathways independent of coagulation. Additional studies are needed to determine the utility of circulating neutrophil activation and NET markers, alone or in concert with established clinical parameters, as objective and reliable prognostic tools in advanced cancer.

## Introduction

Cancer is one of the leading causes of mortality and morbidity worldwide. Predicting survival in patients with advanced cancer is important because both clinical and personal decisions are affected by life expectancy^1^. Studies have shown that physicians' survival estimates are unreliable and overly optimistic^2,3^, which could lead to unnecessary interventions toward the end of life. Objective prognostic tools are therefore needed to assist clinicians in assessing prognosis in patients with advanced cancer. Various prognostic assessment tools for terminal cancer patients have been proposed^4–7^, but few are used in clinical practice.

Cancer induces a hypercoagulable state that increases with cancer progression and tumor burden^8^ and increases the risk of thromboembolic complications^9,10^. Several studies have proposed that various coagulation and fibrinolysis markers in plasma can be used to predict thrombotic complications^11,12^, clinical outcome^13^ and mortality^14–17^ in cancer patients. D-dimer has previously been shown to be an independent prognostic factor for mortality in patients with a wide range of malignancies^14,18–20^. High levels of extracellular vesicle tissue factor (EV TF) activity have also been associated with poor prognosis in cancer patients^21–23^. In breast cancer, there is an association between plasminogen activator inhibitor type 1 (PAI-1) and poor prognosis^13,15^.

Neutrophil extracellular traps (NETs) are chromatin structures released from neutrophils in response to a variety of stimuli. Although first described as part of our innate immunity and induced by microbial pathogens, several other “NET inducers” have been proposed, including a plethora of factors also associated with the tumor microenvironment, including soluble P-selectin (sP-selectin)^24^, interleukin-8 (IL-8)^25^, and granulocyte-colony stimulating factor (G-CSF)^26^. High levels of IL-8^27,28^, sP-selectin^17,29,30^ and the neutrophil activation marker neutrophil elastase (NE)^27,31^, are prognostic in patients with cancer. We and others recently showed that markers of neutrophil activation, including citrullinated histone H3 (H3Cit), a marker of NETs, are associated with poor clinical outcome in patients with terminal cancer^27,32^. NETs have also been shown to contribute to enhanced coagulation^33^, a well-known phenomenon in cancer patients^34^.

The objective of this study was to extend our recent findings^27^ showing a prognostic value of plasma H3Cit in terminal cancer by measuring neutrophil activation markers, NET markers and proposed NET inducers, as well as markers of coagulation and fibrinolysis in terminal cancer. In line with our recent study^27^, we found that markers of neutrophil activation and NETs were prognostic in terminal cancer patients. Contrary to our hypothesis, markers of coagulation and fibrinolysis did not have a prognostic value and there were weak or no correlations between these markers and markers of neutrophil activation and NETs.

## Methods

### Study population

One-hundred and six cancer patients were prospectively recruited at the palliative care unit at Stockholms sjukhem, Stockholm, Sweden, between October 2016 and May 2018. Inclusion criteria were active cancer, defined as diagnosis < 1 year and/or disseminated disease, intact cognition and the ability to understand spoken and written Swedish. Inclusion was conducted consecutively twice-weekly when research personnel were available. There were no exclusion criteria. Information on demographic data, comorbidity and on-going medical treatment was collected from hospital records.

Median age was 73 years (IQR 66–81) and 38 patients were men (36%) in the terminal cancer group. The most frequent tumor sites were breast (18%), prostate (16%) and lung (13%), and 89% of patients had known metastatic solid cancer. Demographic data, comorbidity and cancer sites are presented in Table 1. Median survival was 31 days, and 8 (7.5%) patients were alive at the end of the observation period of 180 days. A survival curve of the patient population over 180 days is presented as Supplementary Fig. S1.

**Table 1:** Demographic data, comorbidity and cancer types of study participants.

Baseline characteristics	Cancer patients (n = 106)	Healthy individuals (n = 31)
Age, median (IQR), y	73 (66–81)	72 (71–73)
Male sex, No. (%)	38 (36)	24 (77)
Hypertension, No. (%)	48 (45)	9 (29)
Prior VTE, No. (%)	25 (24)	0
AF, No. (%)	15 (14)	2 (6.5)
DM2, No. (%)	13 (12)	1 (3.2)
Prior stroke, No. (%)	13 (12)	0
COPD, No. (%)	12 (11)	0
CHF, No. (%)	7 (6.6)	0
Acute infection, No. (%)	6 (5.7)	0
Asthma, No. (%)	5 (4.7)	2 (6.5)
Atherosclerosis, No. (%)	4 (3.8)	1 (3.2)
IHD, No. (%)	3 (2.8)	2 (6.5)
RI, No. (%)	3 (2.8)	0
Liver disease, No. (%)	2 (1.9)	0
Neutrophil count****, median (IQR), 10^9^/L	7.9 (4.8–13)	
White blood cell count, median (IQR), 10^9^/L	9.5 (6.6–14)	
Platelet count, median (IQR), 10^9^/L	284 (171–424)	
Hemoglobin, median (IQR), g/L	116 (102–136)	
Cancer type*		
Breast, No. (%)	19 (18)	
Prostate, No. (%)	17 (16)	
Lung, No. (%)	14 (13)	
Colorectal, No. (%)	13 (12)	
Gynecologic, No. (%)	10 (9.4)	
Upper GI tract (excluding pancreatic), No. (%)	9 (8.5)	
Urinary tract, No. %	8 (7.5)	
Pancreatic, No. (%)	7 (6.6)	
CNS, No. (%)	5 (4.7)	
Head and neck, No. %	4 (3.8)	
Multiple myeloma, No. %	4 (3.8)	
Melanoma, No. (%)	2 (1.9)	
Other**, No. (%)	5 (4.7)	
Adenocarcinoma***	75 (72)	

*VTE* venous thromboembolism, *AF* atrial fibrillation, *DM2* diabetes mellitus 2, *COPD* chronic obstructive pulmonary disease, *CHF* chronic heart failure, *IHD* ischemic heart disease, *RI* renal insufficiency, *CNS* central nervous system, *GI* gastrointestinal.

*The total number exceeds 106, as some patients had more than one primary tumor.

**Other cancer types were chronic lymphatic leukemia (n = 2), germinoma (n = 1), sarcoma (n = 1) and neuroendocrine tumor (n = 1). ***Two patients could not be classified as adenocarcinoma or non-adenocarcinoma.

****Neutrophil count was available in 103 patients.

31 healthy and age-matched individuals were included as controls. Exclusion criteria were active or prior cancer diagnosis. Median age was 71 years (IQR 71–73) and 24 individuals were men (77%) in the healthy control group.

All procedures were in accordance with the declaration of Helsinki. All patients and healthy controls signed written informed consent. The study was approved by the regional ethical review board in Stockholm (Dnr 2015/1533–31/1, 2016/359–32, 2016/1102–32, 2016/2051–32/1, 2017/1837–32 and 2018/1845–32/1).

### Laboratory analyses

Venous blood sampling was performed once at study inclusion. Platelet-poor plasma was prepared from citrated whole blood following immediate centrifugation for 20 min at 2000 × g at room temperature, after which they were stored at − 80 °C until further analyses. Samples were thawed on ice at the time of analyses.

Circulating markers were measured as follows: NE using the PMN Elastase Human ELISA Kit (Abcam), sP-selectin using the human sP-selectin/CD62P ELISA Kit (R&D Systems), IL-8 using the V-Plex Human IL-8 Kit (Meso Scale Diagnostics), G-CSF using the Quantikine Human G-CSF Immunoassay (R&D Systems), thrombin antithrombin complexes (TAT) using the Enzygnost TAT micro (Siemens), D-dimer using the Asserachrom D-DI (Diagnostica Stago) and PAI-1 using the Human PAI-1 Activity ELISA Kit (Molecular Innovations), all according to the manufacturers’ instructions.

Nucleosomal H3Cit (H3Cit-DNA) was quantified using a recently validated and highly specific in-house capture ELISA^35^. Briefly, microplates were coated with a monoclonal anti-histone H3 (citrulline R8) antibody (Abcam, Cat# 232,939) and a monoclonal anti-DNA antibody (Cell Death ELISA^PLUS^, Roche) was used for detection. A standard curve was generated by semi-synthetic recombinant nucleosomes, containing citrulline at the 2, 8 and 17th arginine residue of the N-terminal of histone H3. (EpiCypher, Cat#16–1362).

The EV TF activity assay was performed as described previously^36^.

### Statistical analyses

Shapiro–Wilk normality test was used to test for normal and lognormal distributions,. Standard deviations are reported for parametric data, and medians and interquartile ranges for non-parametric data. The student *t* test was used for parametric data, and the Mann Whitney U test for non-parametric data. The Fisher exact test was used to compare proportions. Correlations were investigated with Spearman rank correlation.

Cox regression analyses were performed including the circulating markers as continuous variables. Univariate and multivariate Cox proportional hazards models determined the hazard ratio (HR) of death. In multivariate analysis, adjustment was made for age, sex, metastatic disease and medical treatment (oral anticoagulants, low molecular weight heparins [LMWHs] and corticosteroids). Stata 16 (StataCorp, Houston, TX, USA) was used for Cox models. All remaining statistical analyses were performed using GraphPad Prism 8 (GraphPad Software, Inc., La Jolla, CA, USA). A two-sided *P* value of < 0.05 was considered statistically significant.

## Results

### Markers of neutrophil activation and NETs are elevated and prognostic in patients with terminal cancer

**Figure 1:**
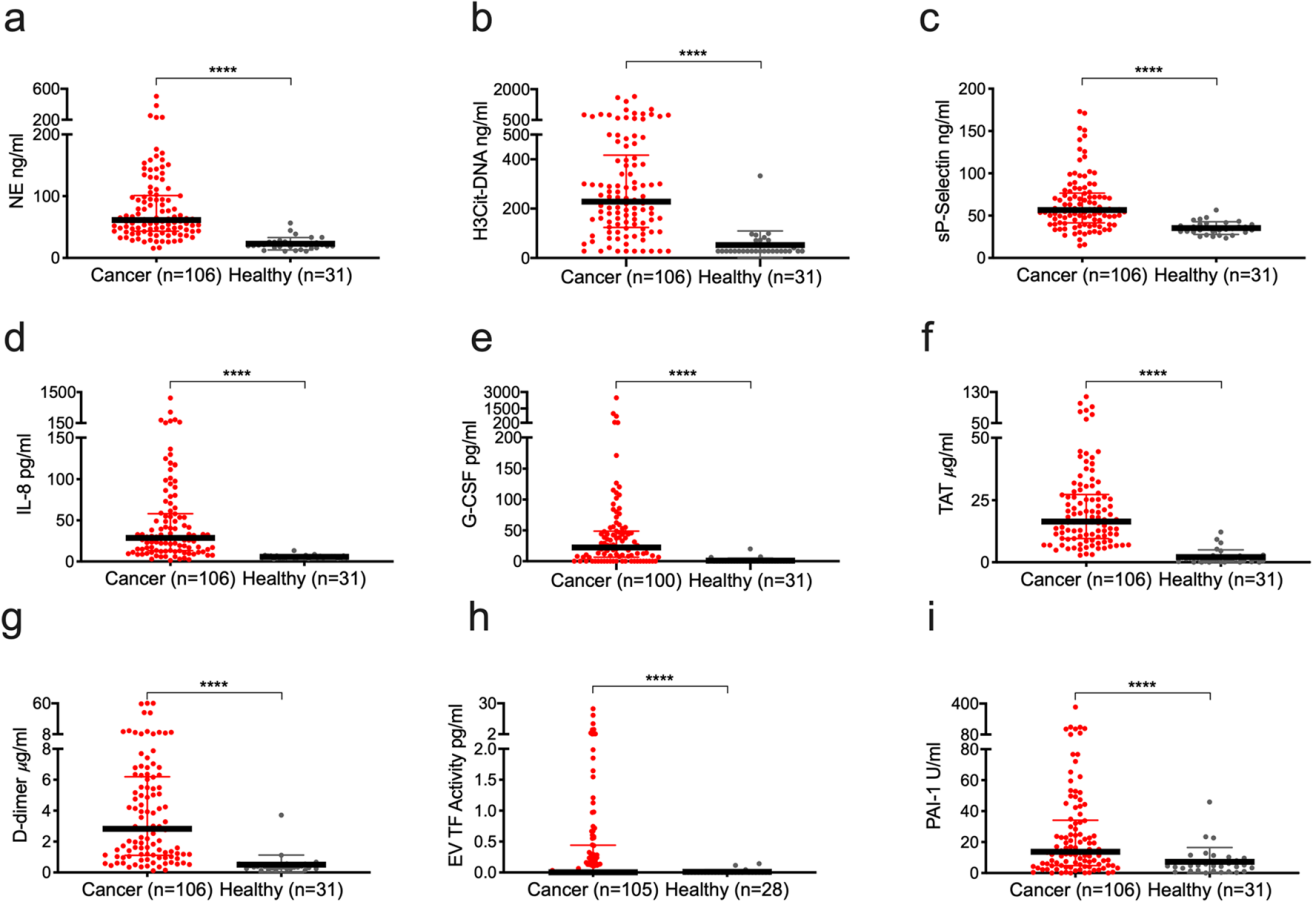
Plasma levels of markers of neutrophil activation, NETs, coagulation and fibrinolysis are elevated in patients with terminal cancer compared to healthy individuals. Lines represent median with IQR. Groups were compared with the Mann–Whitney U test. NS *P* > 0.05, *P < 0.05, **P < 0.01, ***P < 0.001, ****P < 0.0001.

**Figure 2:**
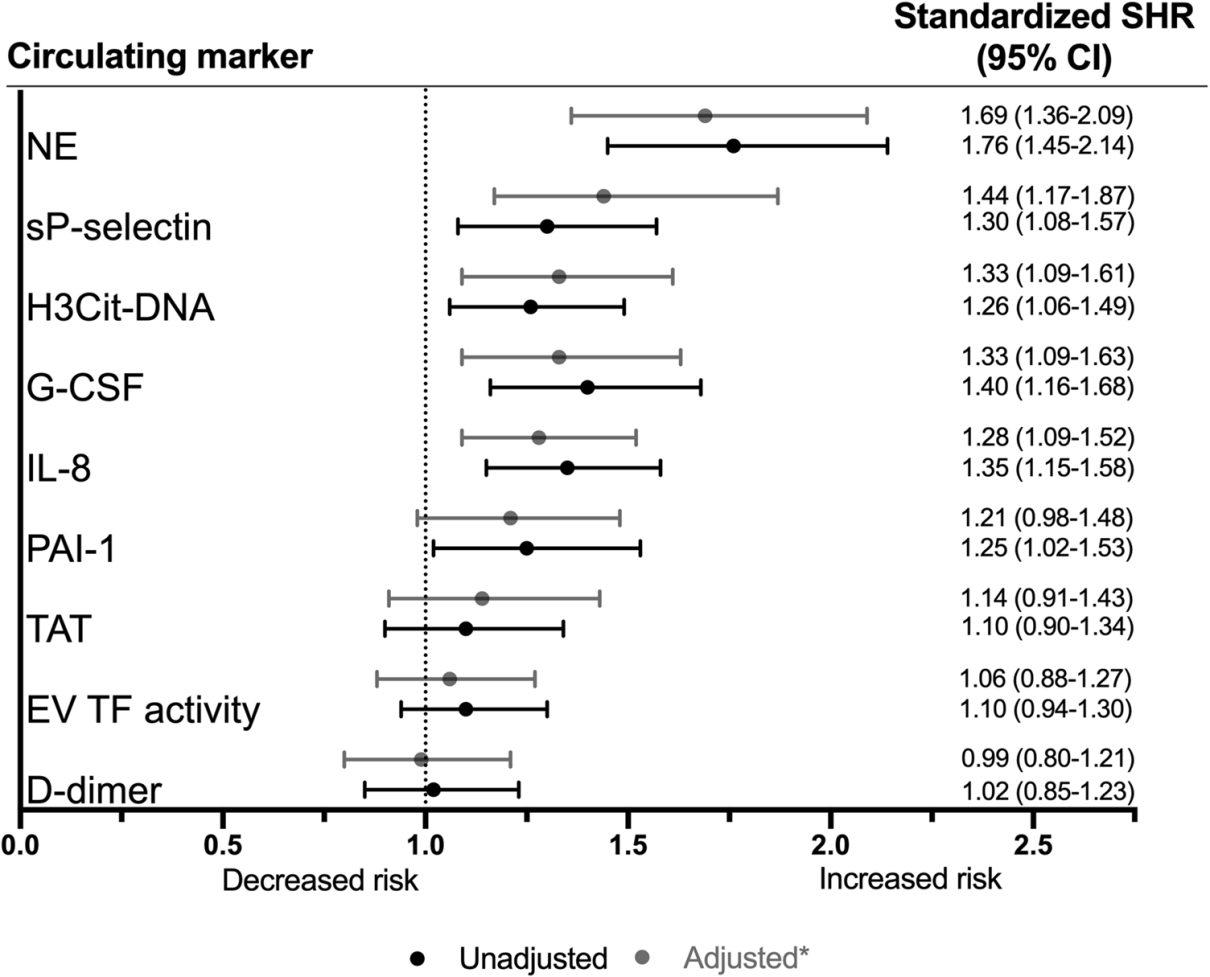
Forest plot of circulating markers as predictors of mortality risk. Calculated in uni- and multivariable Cox proportional hazard models. Comparability between markers is achieved by transforming all variables on a common scale with a mean of zero and standard deviation of I (Z-standardization). The standardized subdistribution hazard ratio (SHR) of each variable can then be interpreted as the multiplicative increase in mortality risk for I standard deviation increase in the variable. HR, hazard ratio; CI, confidence interval; sP-selectin, soluble P-selectin; IL-8, interleukin-8; H3Cit, citrullinated histone H3; NE, Neutrophil elastase; TAT, Thrombin-antithrombin complex; EV TF activity, extracellular vesicle tissue factor activity; PAI-1, plasminogen activator inhibitor-1 activity; G-CSF granulocyte colony-stimulating factor. *Adjusted for age, sex, metastatic disease and medical treatment (oral anticoagulants, low molecular weight heparins [LMWHs] and corticosteroids).

To assess the degree of neutrophil activation and NET formation in patients with terminal cancer, we quantified markers of neutrophil activation (NE), NET formation (H3Cit-DNA) as well as proposed NET inducers (sP-selectin, IL-8 and G-CSF). NE (threefold median increase), H3Cit-DNA (eightfold median increase), sP-selectin (1.7-fold median increase), IL-8 (fivefold median increase) and G-CSF were all elevated in patients with terminal cancer compared to healthy controls (Fig. 1a–e). Elevated levels of H3Cit-DNA were seen in all investigated tumor types (Supplementary Tables S1 and S2). NE and H3Cit-DNA showed moderate correlations with sP-selectin and IL-8, but not with G-CSF (Table 2). We found a strong correlation between plasma H3Cit-DNA levels and the neutrophil activation marker NE (Table 2). The neutrophil activation marker NE and the NET marker H3Cit-DNA were both associated with poor prognosis in uni- and multivariate Cox regression (Fig. 2 and Supplementary Table S3). Furthermore, the proposed NET inducers sP-selectin, IL-8 and G-CSF were also strongly associated with prognosis in Cox regression (Fig. 2 and Supplementary Table S3). To enable comparison, variables were Z-transformed. The marker with the strongest association to poor prognosis in multivariate analysis with adjustments for age, sex, metastatic disease and medical treatments (oral anticoagulants, low molecular weight heparins [LMWHs] and corticosteroids) was NE, followed by sP-selectin, H3Cit-DNA and G-CSF.

**Table 2:** Correlations between markers of neutrophil activation, NETs, coagulation, fibrinolysis in patients with terminal cancer using Spearman R.

	sP-selectin	IL-8	H3Cit-DNA	NE	TAT	D-dimer	PAI-1	G-CSF	EV TF Activity
sP-selectin	1	0.320*	0.390*	0.487*	0.409*	0.304*	0.206*	-0.122	0.465*
IL-8	0.320*	1	0.228*	0.387*	0.065	0.273*	0.321*	0.187	0.373*
H3Cit-DNA	0.390*	0.228*	1	0.377*	0.172	0.235*	0.219*	-0.066	0.144
NE	0.487*	0.387*	0.377*	1	0.354*	0.243*	0.282*	0.037	0.388*
TAT	0.409*	0.065	0.172	0.354*	1	0.450*	0.106	0.028	0.287*
D-dimer	0.304*	0.273*	0.235*	0.243*	0.450*	1	-0.001	0.005	0.207*
PAI-1	0.206*	0.321*	0.219*	0.282*	0.106	-0.001	1	0.285*	0.265*
G-CSF	-0.122	0.187	-0.066	0.037	0.028	0.005	0.285*	1	0.033
EV TF Activity	0.465*	0.373*	0.144	0.388*	0.287*	0.207*	0.265*	0.033	1

*sP-selectin* soluble P-selectin, *IL-8* interleukin-8, *H3Cit* citrullinated histone H3, *NE* Neutrophil elastase, *TAT* Thrombin-antithrombin complex, *EV TF activity* extracellular vesicle tissue factor activity, *PAI-1* plasminogen activator inhibitor-1 activity, *G-CSF* granulocyte colony-stimulating factor. * *P* < 0.05.

### Markers of coagulation and fibrinolysis are elevated but not prognostic in patients with terminal cancer

To investigate coagulation and fibrinolysis in patients with terminal cancer, we quantified procoagulant and anti-fibrinolytic circulating markers as well as markers of coagulation and fibrinolysis (EV TF activity, PAI-1, TAT, and D-dimer). EV TF activity, PAI-1 (threefold median increase), TAT (12-fold median increase), and D-dimer (sevenfold median increase), were all elevated in patients with terminal cancer compared to healthy individuals (Fig. 1f–i). Contrary to our hypothesis, none of the coagulation and fibrinolysis markers were associated with poor prognosis in multivariate Cox regression (Fig. 2). Scatterplots showing plasma levels of markers of neutrophil activation, NETs, coagulation and fibrinolysis plotted against time to death are shown in Supplementary Fig. S2A–I.

### Association between markers of neutrophil activation, NETs, coagulation and fibrinolysis

To assess a possible association between neutrophil activation, NETs and coagulation/fibrinolysis, we compared correlations between NE and H3Cit-DNA with TAT, D-dimer, EV TF activity and PAI-1 (Table 2). NE and H3Cit-DNA were weakly but significantly correlated with D-dimer and PAI-1. NE, but not H3Cit-DNA, correlated with EV TF activity and TAT.

### Medical treatment

Since there were no exclusion criteria, patients received various medical treatments, as would be expected in this patient population. Three of the 106 patients were administered oral anticoagulants (OAC; direct oral anticoagulants or warfarin), and 37 were administered LMWHs. The different levels of circulating markers for different categories according to medical treatment is presented in supplementary Table S4. There was no difference in plasma biomarker levels between patients with prophylactic dose of LMWH compared to patients without anticoagulant treatment. Patients with a treatment dose of either LMWH or OACs had lower TAT levels and a trend towards lower D-dimer levels compared to patients without anticoagulant treatment (*P* = 0.007 and *P* = 0.064, respectively). Patients with corticosteroids had lower IL-8 and PAI-1 levels compared to patients without corticosteroids (*P* = 0.028 and *P* = 0.027, respectively).

## Discussion

This study shows a clear association of markers of neutrophil activation and NETs with poor prognosis in patients with terminal cancer. We used a new and rigorously validated assay for measuring circulating H3Cit-DNA^35^, with results similar to our previous study^27^. Interestingly, we did not find an association between markers of coagulation and fibrinolysis and poor prognosis, despite the well-known association between cancer and thrombosis. There were furthermore weak or no associations between the NET marker H3Cit-DNA and markers of coagulation and fibrinolysis, suggesting that neutrophils and NETs may play a role in poor clinical outcome independently of coagulation and fibrinolysis in these patients.

We found a strong correlation between plasma H3Cit-DNA levels and neutrophil activation marker NE. Since NET formation has been implicated in a wide range of malignancies^37–40^, and histone citrullination is considered key for NET formation^41–43^, neutrophil activation and NET formation thereby seem to be a likely source of circulating H3Cit-DNA. H3Cit-DNA levels furthermore correlated strongly with the platelet and endothelial activation marker sP-selectin. This correlation could either be due to direct platelet activation leading to NET formation^44^, sP-selectin inducing NET formation through P-selectin/PSGL-1 interaction^24^, or a consequence of NET formation, as NETs have been shown to capture and activate platelets^45^. IL-8 has also been shown to induce NET formation^25^, and, accordingly, H3Cit-DNA levels correlated with IL-8 levels. We cannot, however, exclude a tumor cell origin of H3Cit-DNA complexes. Peptidylarginine deiminase 4 (PAD4), the enzyme responsible for citrullination of histones, has been shown to be expressed in various tumor cells^46^, and tumor cells expressing PAD4 have furthermore been shown to extrude NET-like structures containing citrullinated histones, including H3Cit^42^. Notably, in vitro experiments have shown that heparins remove histones from the chromatin backbone, leading to destabilization of NETs^45^. Neither prophylactic nor treatment dose of LMWH were associated with levels of H3Cit-DNA in our study. However, the circulating concentration of LMWH is likely lower than the concentrations previously shown to dismantle and destabilize NETs. In the above-mentioned paper, the authors found that a heparin concentration of ≥ 10 μg/ml (equal to ≥ 5000 IU/ml) but not 1 μg/ml dismantled NETs and lead to histone release (detected by immunoblotting). This concentration is likely orders of magnitude higher than the concentrations reached in circulation after administration of 4500 IU of LMWH once daily. In comparison, treatment dose of heparin after acute venous thromboembolism resulted in plasma concentrations of heparin between 0.2 to 0.4 IU/mL (equal to 0.0004 to 0.0008 μg/ml) measured by protamine sulfate neutralization^47^.

The elevations of markers of neutrophil activation, NET formation, coagulation and fibrinolysis in our cancer patients compared to healthy individuals are consistent with prior studies^13,16,21,27,48–61^. Our data extend these findings to patients with terminal cancer, and show that markers of neutrophil activation and NETs are prognostic in our patients with terminal cancer. Since NETs have been proposed to be induced by inflammatory cytokines, the association between high levels of markers NETs and poor prognosis could be due to an inflammatory state, which has been linked to poor prognosis in cancer^62^.

None of the markers of coagulation and fibrinolysis were associated with poor prognosis in our patient population. This contrasts to several previous studies^13–15,18–20,22,23^. However, the patients in our study suffered from terminal cancer, with a median survival of 31 days, compared to a median survival of 264 days or longer in prior studies. In earlier stages of cancer, an activated coagulation indicates a poor prognosis. The majority of terminal cancer patients have an activated coagulation system, which may explain why markers of coagulation and fibrinolysis lack a prognostic significance in this setting.

We were surprised to find weak or no correlations between neutrophil activation and NET markers and markers of coagulation/fibrinolysis, despite numerous studies linking NETs to coagulation^45,63–65^. However, most of these studies employed murine models, and whether intact NETs directly activate the coagulation system is under debate^33^. Purified DNA and histones, but not intact NETs or reconstituted chromatin, have been shown to induce thrombin generation, questioning the procoagulant property of intact NETs^66^. Another recent study observed that intact NETs can occlude small vessels, and in some cases these microthrombi did not contain fibrin or von Willebrand factor^67^. Taken together, these data suggest that NETs may be linked to poor prognosis independently of coagulation, either due to direct endothelial toxicity^44^ or coagulation-independent vascular occlusion^67^. We also observed an association between the neutrophil activation marker NE, but not the NET marker H3Cit-DNA, with TAT and EV TF activity. This suggests a stronger association between neutrophil activation and coagulation compared to the association between NET formation and coagulation in terminal cancer patients. Data from murine models^68,69^, as well as data from smaller studies in humans^70,71^ support the hypothesis that extensive NET formation in cancer patients leads to cancer-associated arterial microthrombosis, presenting as multi-organ failure. In line with the above-mentioned study^66^, these NET-associated microthrombi may indeed be due to a mechanical obstruction rather than an activation of the coagulation cascade, in which case DNase may be an interesting and novel approach to alleviate cancer-associated microthrombosis and multi-organ failure in this patient population.

This study has some limitations. Since this was an exploratory study, the small sample size of the study hampers comparisons between tumor subgroups and associations between circulating markers and venous or arterial thromboembolism. The healthy control group was matched for age but not sex, however, this does not affect the main prognostic findings in the cancer group. Further limitations include possible bias and confounding factors (e.g. treatment). To minimize the bias that comes with dichotomizing continuous variables, we employed Cox regression including the circulating markers as continuous variables.

In conclusion, our results show that neutrophil activation and NET markers, but not markers of coagulation and fibrinolysis, are strongly associated with poor prognosis in patients with terminal cancer. The lack of associations between neutrophil activation and NET markers and markers of coagulation and fibrinolysis furthermore suggest that neutrophils and NETs contribute to a poor prognosis through pathways not directly related to coagulation. Further and larger studies should investigate the potential of neutrophil activation and NET markers in the quest for clinical objective prognostic tools and novel therapeutic targets in patients with advanced cancer.

## Supplementary Information

Supplementary Information
