# Supplementary material for: Prognostic value of circulating markers of neutrophil activation, neutrophil extracellular traps, coagulation and fibrinolysis in patients with terminal cancer
Source: Sci Rep. 2021 Mar 3;11:5074. doi: 10.1038/s41598-021-84476-3 (PMC7930088; doi:10.1038/s41598-021-84476-3)
Supplement: Supplementary file 1 — Supplementary Information [file 41598_2021_84476_MOESM1_ESM.pdf]

Prognostic value of circulating markers of neutrophil activation, neutrophil extracellular traps, coagulation and fibrinolysis in patients with terminal cancer

Axel Rosell[1*], Katherina Aguilera[1], Yohei Hisada[2], Clare Schmedes[2], Nigel Mackman[2], Håkan Wallén[3], Staffan Lundström[4,5], Charlotte Thålin[1]

1. Department of Clinical Sciences, Danderyd Hospital, Division of Internal Medicine, Karolinska Institutet, Stockholm, Sweden
2. UNC Blood Research Center, Division of Hematology, Department of Medicine, University of North Carolina at Chapel Hill, Chapel Hill, North Carolina, USA
3. Department of Clinical Sciences, Danderyd Hospital, Division of Cardiovascular Medicine, Karolinska Institutet, Stockholm, Sweden
4. Palliative Care Services and R&D-unit, Stockholms Sjukhem Foundation, Stockholm, Sweden
5. Department of Oncology-Pathology, Karolinska Institutet, Stockholm, Sweden

*Corresponding author:
axel.rosell@ki.se
Telephone: +46 73 24 79 615
Adress: Kliniskt Forskningscentrum Norr
Department of Clinical Sciences, Danderyd Hospital, Karolinska Institutet
182 88 Stockholm, Sweden

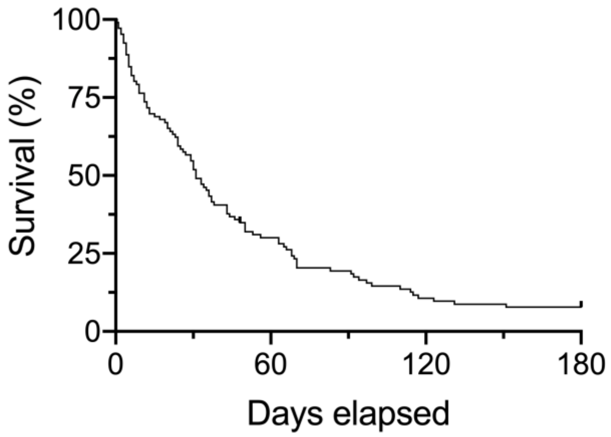

Supplementary Figure S1. Cumulative survival of the 106 patients included in the study over the study period of 180 days.

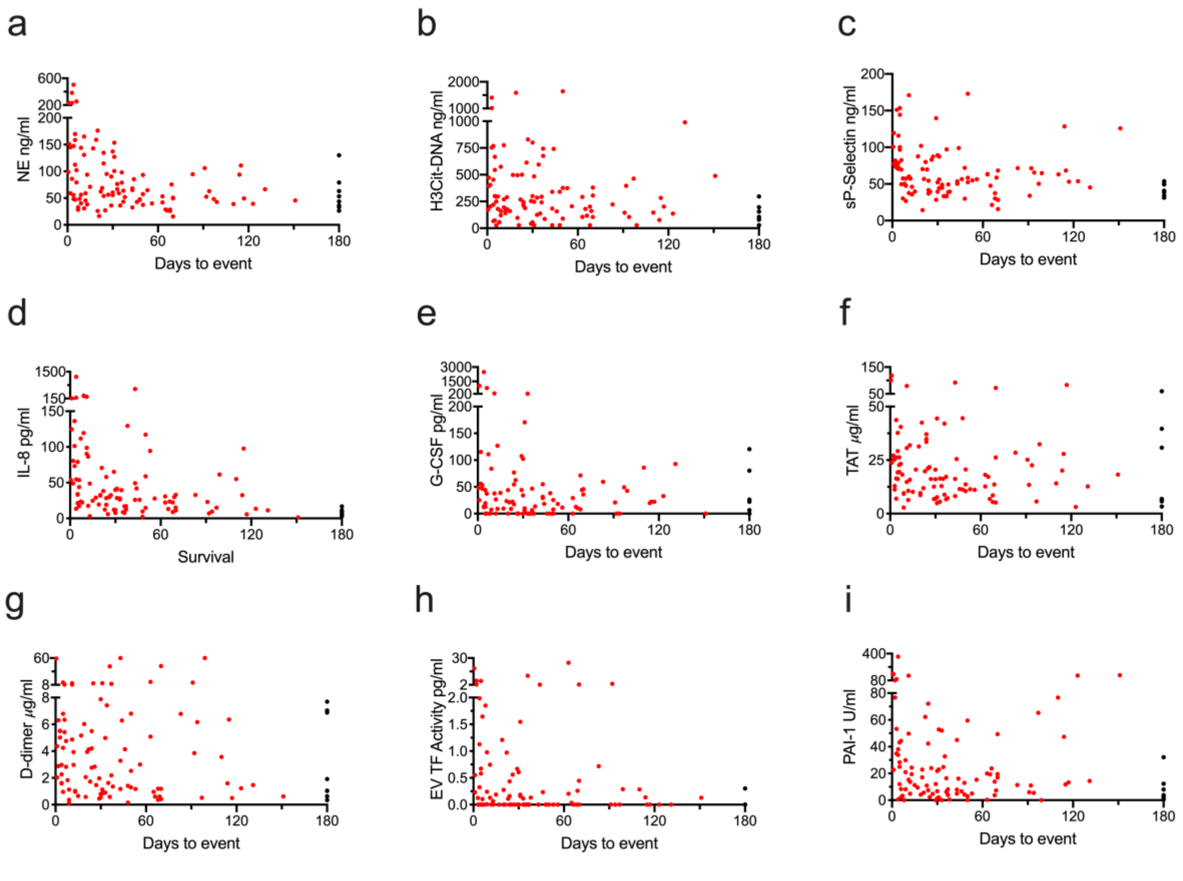

Supplementary Figure S2. Scatterplots showing plasma levels of markers of neutrophil activation, NETs, coagulation and fibrinolysis and days to event. Red dots represent deceased patients and black dots represent patients alive at the end of the observation period of 180 days.

Supplementary Table S1. Levels of circulating markers for different cancer types.

	Upper GI tract (n=9)	Lung (n=14)	Colorectal (n=13)	Melanoma (n=2)	Breast (n=19)	Gynecologic (n=10)	Prostate (n=17)	CNS (n=5)	Pancreatic (n=7)	Urinary tract (n=8)	Head and neck (n=4)	Multiple myeloma (n=4)	Other* (n=5)
sP-selectin, median (IQR), ng/ml	50 (44-71)	66 (54-89)	66 (50-93(	48 (47-49)	56 (42-71)	56 (50-76)	63 (54-87)	41 (33-48)	73 (54-81)	54 (51-74)	34 (32-35)	48 (41-52)	46 (31-68)
IL-8, median (IQR), pg/ml	33 (26-54)	22 (17-71)	37 (28-84)	26 (15-38)	30 (14-42)	26 (19-44)	33 (18-61)	9.0 (8.6-13)	38 (18-70)	28 (19-54)	9.4 (6.8-11)	20 (8.1-92)	3.0 (2.9-12)
H3Cit-DNA, median (IQR), ng/ml	161 (157-248)	245 (104-300)	270 (225-678)	181 (179-182)	296 (105-545)	429 (205-555)	187 (141-300)	180 (136-202)	249 (106-268)	357 (127-575)	142 (76-207)	157 (146-165)	80 (42-178)
NE, median (IQR), ng/ml	52 (48-85)	63 (53-135)	92 (79-97)	35 (34-36)	67 (56-129)	55 (45-123)	46 (38-113)	33 (29-36)	59 (52-71)	69 (63-114)	45 (34-64)	58 (40-76)	44 (38-47)
TAT, median (IQR), μg/ml	12 (9.5-18)	21 (11-38)	17 (13-25)	12 (10-14)	22 (13-26)	19 (7.5-26)	19 (12-42)	16 (5.7-17)	18 (10-35)	16 (15-21)	7.9 (6.9-17)	15 (11-20)	16 (6.8-28)
D-dimer, median (IQR), μg/ml	1.8 (1.1-4.1)	1.6 (1.0-4.4)	3.8 (1.1-5.5)	2.2 (1.6-2.7)	3.0 (1.2-6.3)	2.5 (1.0-4.6)	4.4 (3.6-11)	0.63 (0.50-1.0)	3.0 (2.4-4.5)	1.8 (1.4-9.0)	0.81 (0.54-2.5)	0.82 (0.55-2.7)	0.45 (0.35-1.03)
PAI-1, median (IQR), U/ml	12 (6.6-21)	7.4 (5.5-20)	41 (13-69)	14 (11-17)	17 (5.8-34)	17 (8-60)	19 (6.6-50)	19 (13-33)	3.7 (1.5-10)	5.5 (4.8-14)	9.1 (1.3-20)	13 (6.4-22)	12(8.0-13)
G-CSF, median (IQR), pg/ml	14 (7.4-39)	17 (0-71)	45 (14-64)	91 (73-109)	26 (1.6-44)	25 (9.4-54)	33 (18-46)	13 (0-22)	8.0 (0-10)	19 (11-25)	45 (40-65)	64 (33-95)	44 (22-80)
EV TF Activity, median (IQR), pg/mL	0.19 (0.13-1.1)	0.16 (0-0.67)	0 (0-0.44)	0 (0-0)	0.07 (0-0.36)	0 (0-0.17)	0.29 (0-0.58)	0 (0-0)	0.75 (0-1.3)	0 (0-0.23)	0 (0-0)	0.12 (0-0.25)	0 (0-0)

CNS, central nervous system; GI, gastrointestinal; sP-selectin, soluble P-selectin; IL-8, interleukin-8; H3Cit, citrullinated histone H3; NE, Neutrophil elastase; TAT, Thrombin-antithrombin complex; EV TF activity, extracellular vesicle tissue factor activity; PAI-1, plasminogen activator inhibitor-1 activity; G-CSF granulocyte colony-stimulating factor. *Other cancer types were chronic lymphatic leukemia (n=2), germinoma (n=1), sarcoma (n=1) and neuroendocrine tumor (n=1).

Supplementary Table S2. Levels of circulating markers according to tumor characteristics and for healthy individuals.

	**All cancer patients (n=106)**	**Healthy (n=31)**	**Adenocarcinoma* (n=75)**	**Non-adenocarcinoma (n=29)**	**Localized disease (n=12)**	**Spread disease (n=94)**
sP-selectin, median (IQR), ng/ml	57 (41-77)	34 (31-40)	59 (46-82)	50 (37-57)	52 (46-74)	57 (42-57)
IL-8, median (IQR), pg/ml	29 (13-58)	5.5 (4.5-6.6)	31 (18-55)	15 (9.0-71)	11 (8.9-43)	30 (16-57)
H3Cit-DNA, median (IQR), ng/ml	228 (124-417)	28 (28-62)	254 (151-478)	180 (86-300)	145 (92-218)	246 (142-461)
NE, median (IQR), ng/ml	61 (43-101)	21 (18-26)	67 (47-107)	49 (36-69)	49 (39-62)	64 (44-105)
TAT, median (IQR), μg/ml	16 (9.6-27)	1.3 (0.23-2.3)	17 (11-27)	16 (6.9-28)	18 (12-34)	16 (9.6-26)
D-dimer, median (IQR), μg/ml	2.8 (1.1-6.2)	0.38 (0.27-0.52)	3.6 (1.2-6.3)	1.6 (0.52-3.9)	1.5 (0.6-4.3)	2.9 (1.2-6.3)
PAI-1, median (IQR), U/ml	14 (4.8-34)	4.7 (1.6-8.8)	16 (4.7-41)	12 (5.9-30)	13 (5.1-22)	14 (4.9-37)
G-CSF, median (IQR), pg/ml	22 (6.5-49)	0 (0-0)	23 (6.4-47)	24 (13-55)	3.9 (0-15)	26 (8.9-50)
EV TF Activity, median (IQR), pg/mL	0 (0-0.44)	0 (0-0)	0.13 (0-0.64)	0 (0-0.11)	0 (0-0.36)	0.08 (0-0.44)

sP-selectin, soluble P-selectin; IL-8, interleukin-8; H3Cit, citrullinated histone H3; NE, Neutrophil elastase; TAT, Thrombin-antithrombin complex; EV TF activity, extracellular vesicle tissue factor activity; PAI-1, plasminogen activator inhibitor-1 activity; G-CSF granulocyte colony-stimulating factor. *Two patients could not be classified as adenocarcinoma or non-adenocarcinoma.

Supplementary Table S3. Comparison of Z-scores of H3Cit-DNA and other circulating markers as predictors of mortality risk.

	**Unadjusted** Univariable SHR (95% CI)	***P***	**Model 1** Multivariable SHR (95% CI)	***P***	**Model 2** Multivariable SHR (95% CI)	***P***	**Model 3** Multivariable SHR (95% CI)	***P***	**Model 4** Multivariable SHR (95% CI)	***P***
sP-selectin	1.30 (1.08-1.57)	0.005	1.36 (1.12-1.66)	0.002	1.39 (1.13-1.70)	0.001	1.40 (1.14-1.72)	0.001	1.44 (1.17-1.87)	0.001
IL-8	1.35 (1.15-1.58)	<0.001	1.33 (1.13-1.57)	0.001	1.31 (1.11-1.56)	0.001	1.32 (1.12-1.55)	0.001	1.28 (1.09-1.52)	0.003
H3Cit-DNA	1.26 (1.06-1.49)	0.01	1.28 (1.06-1.55)	0.011	1.28 (1.05-1.56)	0.013	1.32 (1.09-1.60)	0.005	1.33 (1.09-1.61)	0.005
NE	1.76 (1.45-2.14)	<0.001	1.76 (1.44-2.15)	<0.001	1.75 (1.42-2.16)	<0.001	1.72 (1.41-2.11)	<0.001	1.69 (1.36-2.09)	<0.001
TAT	1.10 (0.90-1.34)	0.37	1.16 (0.94-1.43)	0.16	1.11 (0.89-1.39	0.37	1.21 (0.98-1.49)	0.084	1.14 (0.91-1.43)	0.25
D-dimer	1.02 (0.85-1.23)	0.82	1.02 (0.84-1.24)	0.85	0.99 (0.81-1.20)	0.89	1.03 (0.84-1.26)	0.78	0.99 (0.80-1.21)	0.89
EV TF activity	1.10 (0.94-1.30)	0.24	1.08 (0.91-1.29)	0.39	1.10 (0.92-1.32)	0.29	1.05 (0.87-1.26)	0.60	1.06 (0.88-1.27)	0.56
PAI-1	1.25 (1.02-1.53)	0.032	1.22 (0.99-1.50)	0.058	1.23 (1.00-1.51)	0.055	1.21 (0.99-1.49)	0.063	1.21 (0.98-1.48)	0.07
G-CSF	1.40 (1.16-1.68)	<0.001	1.38 (1.14-1.67)	0.001	1.37 (1.13-1.66)	0.001	1.36 (1.12-1.65)	0.002	1.33 (1.09-1.63)	0.005

Calculated in uni- and multivariable Cox proportional hazard models. Comparability between markers is achieved by transforming all variables on a common scale with a mean of zero and standard deviation of I (Z-standardization). The standardized subdistribution hazard ratio (SHR) of each variable can then be interpreted as the multiplicative increase in mortality risk for I standard deviation increase in the variable.

HR, hazard ratio; CI, confidence interval; sP-selectin, soluble P-selectin; IL-8, interleukin-8; H3Cit, citrullinated histone H3; NE, Neutrophil elastase; TAT, Thrombin-antithrombin complex; EV TF activity, extracellular

vesicle tissue factor activity; PAI-1, plasminogen activator inhibitor-1 activity; G-CSF granulocyte colony-stimulating factor.

Model 1: Adjusted for age, sex, metastatic disease. Model 2: Adjusted for age, sex, metastatic disease, oral anticoagulants and low molecular weight heparins (LMWHs). Model 3: Adjusted for age, sex, metastatic disease and corticosteroid treatment. Model 4: Adjusted for age, sex, metastatic disease and medical treatment (oral anticoagulants, low molecular weight heparins [LMWHs] and corticosteroids).

Supplementary Table S4. Levels of circulating markers in different medical treatment groups.

	**LMWH any dose (n=37)**	**LMWH prophylactic dose* (n=16)**	**LMWH treatment dose* or OAC (n=18)**	**No anticoagulant treatment (n=65)**	**Corticosteroids (n=81)**	**No corticosteroids (n=25)**
sP-selectin, median (IQR), ng/ml	57 (39-82)	57 (37-91)	53 (39-74)	57 (45-72)	54 (39-78)	58 (50-72)
IL-8, median (IQR), pg/ml	30 (17-59)	25 (17-94)	26 (14-49)	29 (13-54)	26 (12-54)‡	38 (25-90)
H3Cit-DNA, median (IQR), ng/ml	290 (161-396)	297 (160-457)	279 (152-413)	205 (104-472)	245 (117-463)	203 (157-295)
NE, median (IQR), ng/ml	59 (46-90)	59 (45-105)	53 (42-68)	64 (43-113)	59 (44-95)	71 (43-115)
TAT, median (IQR), μg/ml	13 (8.6-21)	18 (9.2-24)	12 (5.6-18)†	20 (12-31)	18 (9.6-29)	13 (10-23)
D-dimer, median (IQR), μg/ml	2.2 (1.0-4.1)	2.8 (1.8-6.1)	1.6 (0.49-5.1)	3.3 (1.2-6.3)	2.0 (1.0-6.3)	4.1 (2.5-5.5)
PAI-1, median (IQR), U/ml	14 (6.3-52)	11 (4.6-37)	13 (7.2-63)	15 (4.9-28)	12 (4.2-25)‡	23 (7.9-59)
G-CSF, median (IQR), pg/ml	21 (0-52)	7.9 (0-26)	25 (3.2-60)	22 (9.8-48)	21.7 (6.7-45)	38 (7.0-71)
EV TF Activity, median (IQR), pg/mL	0.12 (0-0.56)	0.01 (0-0.31)	0.03 (0-0.97)	0 (0-0.30)	0 (0-0.51)	0 (0.11-0.28)

sP-selectin, soluble P-selectin; IL-8, interleukin-8; H3Cit, citrullinated histone H3; NE, Neutrophil elastase; TAT, Thrombin-antithrombin complex; EV TF activity, extracellular vesicle tissue factor activity; PAI-1, plasminogen activator inhibitor-1 activity; G-CSF granulocyte colony-stimulating factor; DOAC, direct oral anticoagulants; LMWH, Low molecular weight heparins.

*Treatment dose was defined as daily dose of dalteparin ≥ 10 000 IU or tinzaparin ≥ 9 000 IU, and prophylactic doses were ≤ 4500 IU for tinzaparin and ≤ 5000 IU for dalteparin. †Significantly different from no anticoagulant treatment. ‡ Significantly different from no corticosteroid treatment.
